# Genetic Diversity and Population Structure of a Large USDA Sesame Collection

**DOI:** 10.3390/plants13131765

**Published:** 2024-06-26

**Authors:** Damien Seay, Aaron Szczepanek, Gerald N. De La Fuente, Eric Votava, Hussein Abdel-Haleem

**Affiliations:** 1 US Arid Land Agricultural Research Center, USDA ARS, Maricopa, AZ 85138, USA; 2 Sesaco Corporation, 5405 Bandera Rd. San Antonio, TX 78238, USA

**Keywords:** *Sesamum indicum* L., genotyping-by-sequencing (GBS), next-generation sequencing single-nucleotide polymorphism (SNP)

## Abstract

Sesame, *Sesamum indicum* L., is one of the oldest domesticated crops used for its oil and protein in many parts of the world. To build genomic resources for sesame that could be used to improve sesame productivity and responses to stresses, a USDA sesame germplasm collection of 501 accessions originating from 36 countries was used in this study. The panel was genotyped using genotyping-by-sequencing (GBS) technology to explore its genetic diversity and population structure and the relatedness among its accessions. A total of 24,735 high-quality single-nucleotide polymorphism (SNP) markers were identified over the 13 chromosomes. The marker density was 1900 SNP per chromosome, with an average polymorphism information content (PIC) value of 0.267. The marker polymorphisms and heterozygosity estimators indicated the usefulness of the identified SNPs to be used in future genetic studies and breeding activities. The population structure, principal components analysis (PCA), and unrooted neighbor-joining phylogenetic tree analyses classified two distinct subpopulations, indicating a wide genetic diversity within the USDA sesame collection. Analysis of molecular variance (AMOVA) revealed that 29.5% of the variation in this population was due to subpopulations, while 57.5% of the variation was due to variation among the accessions within the subpopulations. These results showed the degree of differentiation between the two subpopulations as well as within each subpopulation. The high fixation index (*F_ST_*) between the distinguished subpopulations indicates a wide genetic diversity and high genetic differentiation among and within the identified subpopulations. The linkage disequilibrium (LD) pattern averaged 161 Kbp for the whole sesame genome, while the LD decay ranged from 168 Kbp at chromosome LG09 to 123 Kbp in chromosome LG05. These findings could explain the complications of linkage drag among the traits during selections. The selected accessions and genotyped SNPs provide tools to enhance genetic gain in sesame breeding programs through molecular approaches.

## 1. Introduction

Sesame, *Sesamum indicum* L., is a 5000-year-old domesticated crop [1]. In many countries, sesame is one of the main industrial crops used for its seed oil and protein. Sesame seed’s oil content reaches 50% of the seed weight, which is higher than that of cotton (15%), soybean (20%), canola (40%), and sunflower (40%). Furthermore, oleic (40%) and linoleic (40%) acids are the prominent fatty acids in sesame oil, making it an ideal healthy fat source in nutritional applications [2]. That said, while oils account for the majority of the seed weight, it is important to note that sesame seed proteins also contribute to 28% of the seed weight [3].

The first step in the genetic enhancement of a crop is to understand its genetic diversity. Advancements in next-generation sequencing technologies have paved the road for a deeper understanding of these relationships. As such, it has been made possible to expedite the discovering of genes and alleles controlling these traits via genome-wide association studies (GWAS), genetic mapping, and omics approaches such transcriptomics, metabolomics, and lipidomics, and the selection of parental accessions for use in conventional plant breeding techniques. As one of the important oil crops, sesame’s germplasm and accessions were characterized both phenotypically [4,5,6,7,8,9] and genetically [10,11,12], indicating the possibility of improving sesame for high productivity and biotic and abiotic stress tolerance [5,13,14]. Sesame’s genetic diversity was assayed using different types of biochemical [15] and molecular markers, among which were random amplified polymorphic DNA (RAPD) [16,17,18], simple sequence repeats (SSR) [4,5,19,20], inter-simple sequence repeats (ISSR) [11,21], amplified fragment length polymorphism (AFLP) [7,22], and sequence-related amplified polymorphism (SRAP) [23]. Utilizing next-generation sequencing (NGS) technologies reduced the genotyping time and effort required to genotype thousands of genotypes and, thus, reduce the cost, which has made single-nucleotide polymorphism (SNP) markers the most common marker type for genome-wide studies [24], where SNPs are biallelic markers that are uniformly distributed throughout the genomes. SNP markers were used to explore the genetic diversity of Mediterranean [12] and Ethiopian [25] sesame core collections. Genotyping-by-sequencing technology (GBS) [26] is one of the NGS high-throughput genotyping technologies that was used for SNP discovery and the genotyping of several crops, such as *Brassica rapa* L. [27], *Brassica carinata* A. Braun [28], *Brassica juncea* L. [29], *Camelina sativa* [30], *Olea europaea* [31], *Glycine max* [32], *Citrullus lanatus* [33], and *Triticum aestivum* [34].

Our current study used GBS technology to genotype the USDA sesame collection and characterize its genetic diversity and population structure. Specifically, the objectives of our study were as follows: to (1) discover SNP markers in the USDA sesame diversity panel; (2) characterize the genetic diversity and population structure; and (3) characterize the genetic differentiation between and within its subpopulations.

## 2. Results and Discussion

### 2.1. Single-Nucleotide Polymorphism Markers Coverage and Polymorphism Analyses

**Figure 1:**
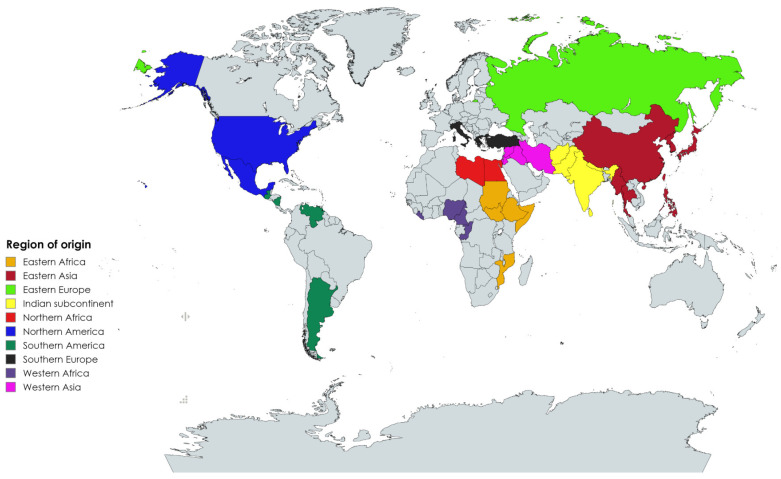
Geographical distribution of the origin of the 501 accessions of sesame used in this study.

Besides playing a crucial role in the evolution of plant populations, the degree of genetic diversity has an important role in improving economic crops through the integration of new traits/alleles/genes into the genetic pool. It is a fundamental step in studying the genetic relationships among and within crop populations to improve their productivity and responses to environments. To reach this goal, phenotypic and genetic diversity studies were conducted to explore the variations in sesame populations [4,5,6,10,12,13,14,15,16,17,18,19,20,21,22,23,25,35,36,37,38]. In this study, we employed NGS-GBS technology to gain a deep understanding of the USDA sesame collection and study the genetic relationships among its accessions. The advantages of using GBS technology to discover SNP markers include genome-wide coverage, a lower error rate, and increased cost effectiveness [39,40]. The USDA sesame collection consists of 501 accessions collected from 36 countries in Asia (245 accessions), Africa (119 accessions), Europe (77 accessions), North America (42 accessions), and South America (18 accessions) (Figure 1, Supplementary Table S1). Previous genetic and cytological studies suggest that the center of origin for sesame is the African continent and Indian subcontinent [41], with some cultivated sesame growing in Asia being able to hybridize with *S. indicum* to produce fertile seeds [1]. Refs. [39,40] indicated that the wide distribution of sesame accessions over a wide area of the world could be due to material exchange around the trade routes.

**Figure 2:**
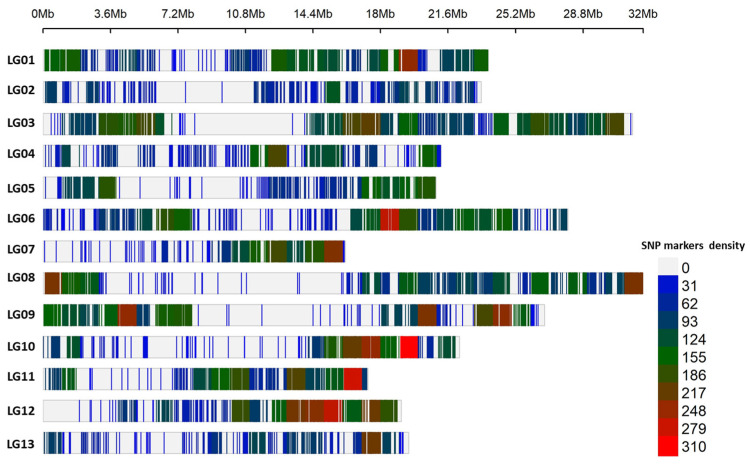
Genomic distributions and marker densities of 24,735 single-nucleotide polymorphisms (SNPs) across the 13 chromosomes of *Sesamum indicum*. Centromere is suggested by white blocks.

The sequencing of the USDA sesame accessions using an NGS-GBS pipeline resulted in 3,277,732,037 raw reads. After trimming off the barcodes and truncating bad sequences, those reads were filtered down to 2,962,283,105 demultiplexed reads, where 881,504 tags were mapped to the aligned chromosome using the *Sesamum indicum* genome sequence (genome assembly: ASM2616843v1) [42]. The GBS pipeline identified 24,735 high-quality bi-allelic SNPs after rejecting SNPs with an MAF < 0.05, a missing rate > 20%, and a heterozygous proportion <25%. The 24,735 SNP markers were uniformly distributed across the 13 sesame chromosomes (Figure 2, Table 1).

In general, there was an increase in polymorphism near the telomeres compared to the centromeres (Figure 2). The average number of SNPs per chromosome was 1903, with chromosome LG03 having the largest number of SNPs (3118), while chromosome LG13 had the fewest mapped SNPs (1198). There was an SNP mapped every 12.76 Kbp, and that coverage ranged from 16.28 Kbp on chromosome LG13 to 9.25 Kbp on chromosome LG12 (Table 1). 

The average SNP density was 81.73 SNPs in an Mbp across the chromosomes (Table 1), and it ranged from 108 SNPs/Mbp mapped on chromosome LG12 to 53 SNPs/Mbp mapped on chromosome LG2 (Table 1). The reasons for the different polymorphism rates among the chromosomes could be related to selection pressure during domestication events and breeding processes and/or different new recombination and mutation rates [28,43,44,45]. The polymorphisms rate is affected by the substitution mutation type and can be classified into transition and transversion mutations. The substitution SNPs mapped to the sesame genome in the current study were 58.4% classified as transition mutations, while 41.6% were classified as transversion mutations (Table 2), which is similar to what is observed in Mediterranean sesames [12], *Brassica rapa* L. [46,47], *B. napus* L. [48], *Brassica juncea* L [29], and *Camelina sativa* L. [30]. One possible explanation for the higher number of transition mutation frequencies relative to transversion mutation frequencies could be the increased stability of transition mutations during natural selection and, consequently, during exposure to selective pressures [30,46,49].

**Figure 3:**
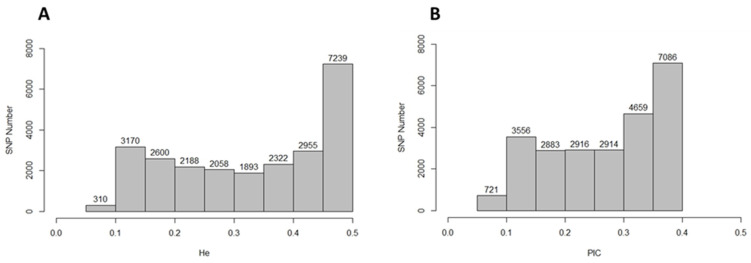
Marker polymorphism and heterozygosity estimations of 24,735 single-nucleotide polymorphism (SNP) markers. (**A**) Expected heterozygosity (He) and (**B**) polymorphic information content (PIC).

Marker polymorphism and heterozygosity estimations are used to determine the usfulness of markers in molecular breeding programs. These estimators are used as well to understand the causes of diversity changes due to mutations and natural and artificial selection forces [50]. For example, a high expected heterozygosity (He), the proportion of heterozygous genotypes expected under the Hardy–Weinberg equilibrium equation [51,52], means larger genetic adaptability and wider popultion genetic diversity. The current results inidcated that the USDA sesame panel has considerable genetic diversity in terms of He, where the He values ranged from 0.088 to 0.500, with an average of 0.332. Approximately 1.3% of the genotyped SNPs had a He ≤ 0.100, while 58% had a He ≥ 0.300 (Figure 3A). These results were slightly lower than the reported He values in sesame [5,38] using a lower number of SSR markers. Perhaps these discrepancies in the He values can be explained by the effects of the population size and diversity as well as the molecular marker types and allele numbers per locus. For example, the He value was 0.283 for rutabaga (*B. napus*) accessions collected from Nordic countries [53], while it was 0.43 for a more diverse group collected from 19 countries from North Africa, Europe, North America, Asia, and New Zealand [48]. Moreover, the He estimation was 0.559 for 48 African sesame genotypes genotyped with 33 SSR markers and 0.11 for 293 African accession genotypes with 6473 SNP markers [25].

The polymorphism information content (PIC), estimates marker polymorphisms and informativeness, is useful tool in linkage analysis and molecular breeding studies [50,54]. The PIC values depend on the marker type, number of alleles, and position of markers on a given chromosome as well as the origin of the population. For example, the PIC values ranged from 0.18 to 0.81 when *Brassica juncea* accessions were genotyped using SSR and RAPD markers, respectively [55], and reached 0.95 [56] with different sets of those markers. The same approach was observed in *Brassica napus* when using RAPD and SSR markers [57,58,59,60] compared to SNP markers [45,48,53]. In the current study, using 501 sesame accesions collected from 36 countries and genotyped with 24,735 SNP markers, the average PIC value was 0.267 (Figure 3B). Previously published results of sesame PIC values indicated that the PIC values ranged from 0.12 using SNP markers [25] to 0.51 using SSR markers [38] for African-origin sesame accessions. Taking into account that SNP markers are biallelic, which reduces the overall PIC values [61], 59.2% and 30.2% of the SNP markers indentified in the current study were considered highly and moderately informative, respectively (Figure 3B). The informativeness of the SNP markers identified using NGS-GBS technology in the current study could lay the foundation for in-depth genome-wide association studies (GWAS) as well as genetic mapping studies to discover alleles/genes related to sesame productivity and response to stresses.

### 2.2. Analysis of Population Structure

**Figure 4:**
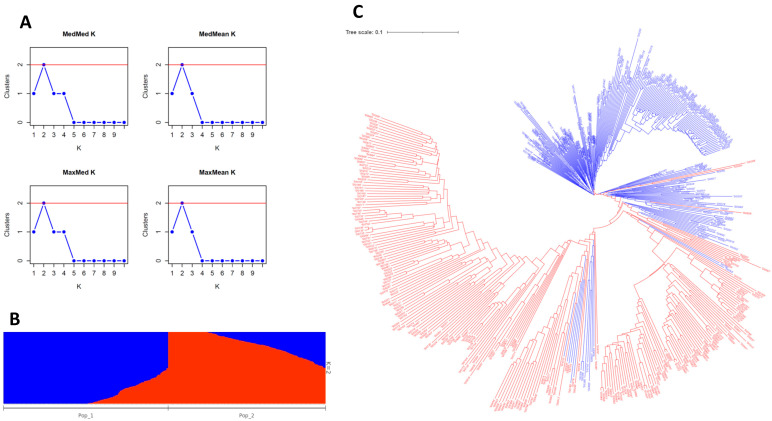
Population structure of the 501 USDA sesame accessions: (**A**) inferred clusters obtained using the Puechmaille method and Structure Selector software, (**B**) estimated population structure based on K = 2 using fastStructure software; and (**C**) the neighbor-joining phylogenetic tree based on genetic distance matrix.

A population structure analysis was conducted to understand the genetic relationships among the panel of 501 sesame accessions. Understanding the population structure of sesame helps to reduce false-positive associations due to the population structure among individuals when conducting GWAS analyses [62]. Further, the classification of subpopulations within the panel can assist with the identification of diverse parental genotypes to be used in future genetic and breeding activities. The maximized marginal likelihood of the fastStructure software was used to group the USDA sesame panel into subpopulations in the range of k = 1 − 10. The MedMedK, MedMeanK, MaxMedK, and MaxMeanK methods [63] (Figure 4A) of the Structure Selector software [64] indicated that the USDA sesame panel was grouped into two distinct subpopulations (Figure 4B; Supplementary Table S1). The number of sesame subpopulations identified in previous studies varied based on the population size, the origin of the genotypes, and the number and type of molecular markers used. For example, previous research has grouped sesame populations into two [7,10,37,38], three [5,12,19,65], or four subpopulations [4,25]. The current results indicated that sesame subpopulation 1 included 256 accessions that were mainly collected from Asia (133 accessions), where 78 accessions were collected from eastern Asia, while subpopulation 2 consisted of 245 accessions, with 121 being collected from Asia (108 from the Indian subcontinent) and 93 being collected from Africa (65 from Sudan) (Supplementary Table S1). It is believed that the origins of cultivated sesame can be traced back to two separate regions, Africa and/or the Indian subcontinent [1,41], from which the worldwide spreading of sesame likely occurred via human migration and trade [12]. This could explain why there are no clear distinguishing features when comparing sesame from different countries.

**Figure 5:**
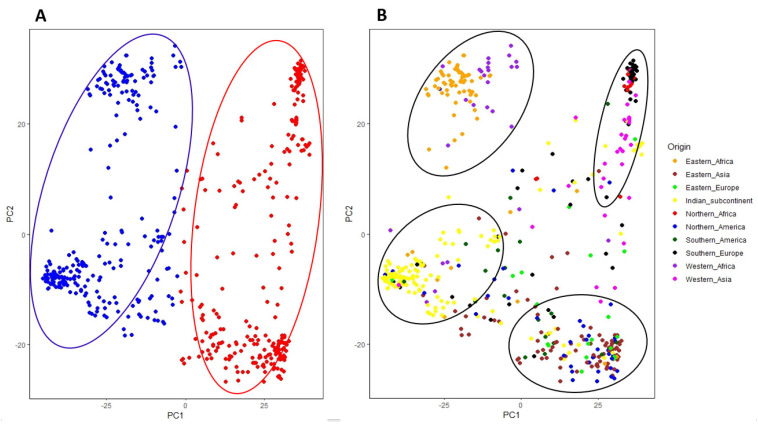
Principal component analysis (PCA) based on (**A**) genetic distance of the two clustered subpopulations and (**B**) genetic distance among accessions and their origin.

To confirm the results of the fastStructure analysis and explore the genetic relatedness among the USDA sesame collection, principal component analysis (PCA) and neighbor-joining (NJ) tree analyses were conducted using the 501 accessions and 24,735 genotyped SNPs. In agreement with the fastStracture results, the phylogenetic analysis showed two major clades with minor displacements of the mixtures (Figure 4C). The clustering patterns in the PCA clearly distinguished two distinct groups (Figure 5A), echoing the fastStructure results. To gain a more in-depth understanding of the relationship among the sesame accessions, the two clusters were reclassified based on their country/continent of origin (Figure 5B). The PCA analysis in respect of the accessions’ origin revealed four subclusters: one derived from the Indian subcontinent, a second from eastern and western Africa, a third from eastern Europe/eastern Asia/North America/South America (Pacific Ocean), and a fourth from southern Europe/western Asia/northern Africa (Mediterranean and Middle East region). Again, these findings support the notion that the modern-day sesame distribution has largely been influenced by historic trade routes and human migration (Figure 5B).

### 2.3. Analysis of Molecular Variance (AMOVA) and Genetic Diversity Indices

An analysis of molecular variance (AMOVA) was conducted to explain the relationship among the two sesame subpopulations and estimate their differentiation. The AMOVA results indicated that 29.5% of the variation in the studied sesame collection was among the subpopulations, 57.5% of the variation was among the accessions within each subpopulation, and the remainder (13%) was attributed to the variation within the accessions (Table 3). The fixation index (*F_ST_*), i.e., the degree of differentiation among the populations, was considered very strongly differentiated at *F_ST_* values were >0.25 [27,66]. Our results showed an *F_ST_* estimate of 0.867, which indicates a very strong differentiation between the two sesame subpopulations.

### 2.4. Linkage Disequilibrium

Understanding the linkage disequilibrium (LD) pattern, the nonrandom associations among alleles at different loci [67,68], can help with exploring the linkage drag, i.e., the unintentional co-selecting of undesirable linked genes [69]. Identifying closely linked polymorphic SNPs led to creating a strong LD and haplotype blocks and, thus, impacted the resolution of the gene identification studies by reducing the recombination rate [28,70,71,72]. Moreover, the LD can explain the genetic diversity, where it is affected by factors that can change the genetic diversity, such as natural selection, population bottlenecks, genetic drift, inbreeding, inversions and gene conversion, the recombination rate, and mating system [73,74].

**Figure 6:**
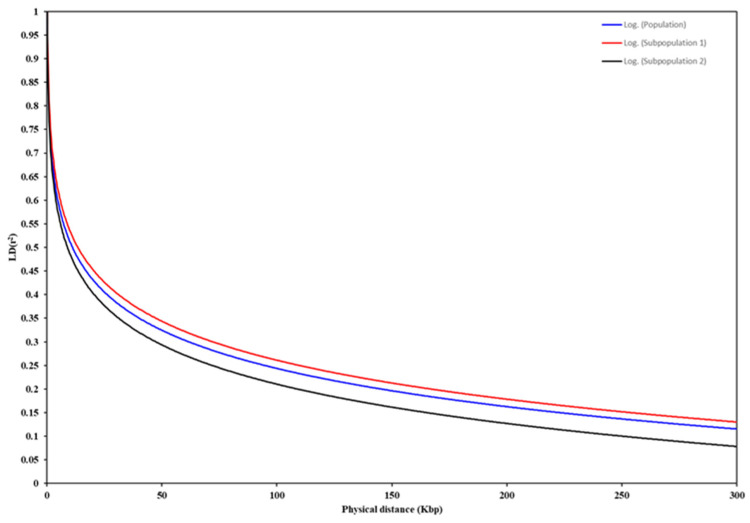
Linkage disequilibrium (LD) decay of the 501 sesame accessions, the r^2^ values plotted against physical distance for whole panel and two subpopulations based on fastStructure software.

The pairwise r^2^, which is the square of the correlation between alleles at two loci in the same gamete, was used to study the LD in the USDA sesame collection mapped with 24,735 SNPs across the 13 chromosomes. The LD decay, at an r^2^ threshold = 0.20, averaged at 160.69 Kbp for the whole sesame genome as a whole population (Figure 6, Table 4). Previous studies have reported different values for the LD decay [10,37,75], controlled by the number and origin of sesame accessions and/or number of SNPs and marker density. In the current study, the LD decay value varied among the subpopulations and chromosomes (Table 4). These results show the variation in the LD decay between subpopulation 1 (166.5 Kbp) and subpopulation 2 (143.8 Kbp) (Figure 6, Table 4). Further, the LD decay varied across the 13 sesame chromosomes, with a value of 123.3 Kbp in chromosome LG05 and a value of 167.7 Kbp estimated at chromosome LG09 (Table 4). The observed variation in the LD decay could be related to differences in polymorphism, including both the number and positions of SNPs as well as diversity among the sesame chromosomes (Figure 2, Table 1). Differences in the LD among chromosomes have previously been reported in other crops. For example, variations in the LD values of brassica chromosomes and sub-genomes were reported to be the result of increased gene conservation, large segmental structural variation [76], and/or the ecogeographical adaptation and artificial selection for important traits [77]; for example, the selection of the flowering time and/or seed quality [78] can reduce genetic diversity and increase the LD and haplotype block size regions. These findings are likely applicable to many domesticated crops, including sesame, and highlight the mechanisms by which the LD variance can be explained in the current study.

### 2.5. Applications of High-Throughput Genotyping of Sesame Accessions

Recent advancements in genomics approaches, such as next-generation sequencing, high-throughput genotyping, and omics technologies, allow plant breeders to explore the genetic diversity within crop populations and, thus, rapidly accelerate plant breeding for germplasm improvement through identifying favorable gene/alleles in specific genotypes. Additionally, the low-cost nature of these methodologies and their overall efficiency have led to the development of advanced, accessible, high-throughput sequencing technologies (HTS) [79]. One of the HTS approaches is genotyping-by-sequencing (GBS) technology, which has facilitated the discovery of hundreds of thousands of SNP markers at a low cost in plant populations [80]. GBS technology was used to characterize the genetic diversity of crops such as *Camelina sativa* [30] and *Brassica juncea* [29], and the identified SNPs have subsequently been used for gene discovery [81,82,83]. As such, these technologies were used to genotype the panel of 501 sesame accessions in this study, collected from different parts of the world and maintained by the USDA-NPGS system, with 24,735 SNP markers. These markers span the entire *Sesamum indicum* genome and illustrate the wide genetic diversity of the panel. Moving forward, the genotyped sesame accessions and the mapped SNP markers will be used to design and conduct allele/gene discovery studies using a GWAS approach, the results of which could lead to the discovery of candidate genes controlling the seed yield, oil content, fatty acid compositions, and tolerances to abiotic (drought, high heat, salt, and flood) and biotic (disease and insect) stresses. Further, phenotyping these accessions may also lead to the identification of genetically diverse accessions with traits of interest. Of these, the genotyped accessions will be used as parents in sesame breeding programs using innovative technologies such as marker-assisted selection and genomic selection, speed breeding, and doubled-haploid methodologies to rapidly increase sesame productivity and stability under different environments.

## 3. Materials and Methods

### 3.1. Plant Materials

The current study assessed a large USDA sesame collection consisting of 501 sesame accessions, including old landraces, old varieties, and breeding lines that were collected from 36 counties in Asia, Africa, Europe, and North and South America (Figure 1, Supplement Table S1) and that is maintained at the USDA-ARS Plant Genetic Resources Conservation Unit (PGRCU), Georgia, USA. The panel was planted in the greenhouse at the U.S. Arid-Land Research Center (ALARC, USDA-ARS) at Maricopa, Arizona, USA, during the summer of 2022. The fresh tissues were collected from one-month-old plants and stored at −80 °C for further analyses.

### 3.2. DNA Extraction and Genotyping-by-Sequencing (GBS)

DNA was extracted according to Muthulakshmi et al. [84] with modifications. Briefly, frozen leaf tissue from each accession (~0.10 g) was lyophilized, and grinding buffer (100 mM Tris-HCl, 5 mM EDTA, 0.35 M sorbitol, 2%PVP, 1% *v*/*v* ßME) was added to the tissue and homogenized using stainless-steel beads and a Geno/Grinder 2010 device (SPEX SamplePrep, Metuchen, NJ, USA). The homogenized tissue was centrifuged, and the polyphenol-/saccharide-containing supernatant was removed. This process was performed two additional times until the supernatant was no longer viscous to ensure the removal of contaminants that could interfere with downstream applications. The remaining steps of the modified CTAB extraction protocol were conducted as described, with DNA being eluted in TE buffer and stored at −20 °C until further use. DNA quality and concentrations were determined using a NanoDrop Spectrophotometer (Thermo Scientific, Boston, MA, USA). Additionally, DNA integrity was determined via BamHI-HF (New England Biolabs, Ipswich, MA, USA) restriction digests, the bands from which were subsequently visualized and assessed after running them in 1% agarose gel electrophoresis. To optimize the GBS library preparation, eight DNA samples were chosen at random to generate GBS libraries with each enzyme, ApeKI, PstI/MspI, PstI/BfaI, NsiI/MspI, and NsiI/BfaI. The libraries were run on an Agilent Tapestation 4200 device (Agilent Technologies, Santa Clara, CA, USA) to observe the fragment sizing and profile. ApeKI was chosen based on its smooth profile and concise fragment size range. The DNA samples were digested with the ApeKI restriction enzyme to prepare the GBS library [26,27,80]. Library preparation and Illumina sequencing were carried out by the University of Wisconsin Bioinformatics Resource Center (UWBRC) using a NovaSeq X Plus 2 × 150 sequencer.

### 3.3. GBS Sequencing and Genotyping Pipeline Analyses

Sequencing and genotyping analyses were carried out according to the UWBRC pipelines and Luo et al. [30] and Abdel-Haleem et al. [29]. Briefly, the raw sequence data, in Fastq formatted files, were trimmed to remove any sequencing adaptors and low-quality bases using skewer software [85]. The pre-processed raw Fastq files were analyzed using the TASSEL v5.0 GBS v2 pipeline [86]. The trimmed GBS sequencing data were converted into a unique tag database using the GBSSeqToTagDBPlugin to trim off the barcodes and truncate the sequences. The GBS tags were exported from the database in fastq format using the TagExportToFastqPlugin and were aligned to the *Sesamum indicum* genome (genome assembly: ASM2616843v1; https://www.ncbi.nlm.nih.gov/datasets/genome/GCA_026168435.1/, accessed on 15 May 2024) using Bowtie 2 [87]. Sequence Alignment Map (SAM)-formatted files were used to import the alignments to the GBS database using the SAMToGBSdbPlugin to determine the potential positions of tags against the reference genome. Identified SNPs were called from the imported alignments using the DiscoverySNPCallerPluginV2. The SNPQualityProfilerPlugin was used to calculate the coverage, depth, and genotypic statistics for alignments in the database. The ProductionSNPCallerPluginV2 was used to convert the SNP data from the fastq format to a Variant Call Format (VCF) file. The mapped SNPs were filtered to keep only biallelic sites with, at most, 20% missing data using vcftools [88].

### 3.4. Population Genetic Analyses

#### 3.4.1. Marker Polymorphism Analyses

The VCF files were converted to HAPMAP format using the TASSEL export feature. The resulted SNPs were further filtered to remove SNPs with a minor allele frequency (MAF) < 0.05. The number of alleles and allele frequencies and MAF for each SNP were calculated using the geno summary function of TASSEL v5.0 GUI [89] (www.maizegenetics.net, accessed on 15 May 2024). The expected heterozygosity (*He*), expressed as the expected proportion of heterozygous genotypes under the Hardy–Weinberg equilibrium, was calculated following Nei’s equation [51], and the polymorphism information content (PIC) was calculated following Botstein et al. [50].

#### 3.4.2. Analysis of Population Structure

The population structure was estimated using the fastStructure software [90] using the Bayesian Markov Chain Monte Carlo (MCMC) model and algorithms that allow for inferring population structures in large SNP data sets. FastStructure was run with the “simple prior” option and the remaining default parameters. The number of subpopulations (K) was set to 1–10, and the best number of subpopulations was selected using the “chooseK.py” function to maximize the marginal likelihood of subpopulations [90]. The MedMedK, MedMeanK, MaxMedK, and MaxMeanK methods [63] of the Structure Selector software [64] were used to identify the subtle clustering patterns and optimal subpopulation number. The admixture proportions of each sesame accession, estimated by fastStructure, were visualized using the Pophelper 2.3.1 software [91].

Principle component analysis (PCA) was carried out using the PCA function of TASSEL v5.0 GUI and plotted using the R package ggplot2 (http://www.r-project.org, accessed on 15 May 2024). An unrooted neighbor-joining phylogenetic tree was constructed using the Tassel software and visualized using interactive tree of life (iTOL) (https://itol.embl.de, accessed on 15 May 2024).

### 3.5. Analysis of Molecular Variance (AMOVA) and Genetic Diversity Indices

The defined two subpopulations determined with the fastStructure and structure selector software were used to conduct an analysis of molecular variance (AMOVA) and to calculate the population fixation index (*F*_ST_) using the Arlequin v.3.52 software [92]. The *F_ST_* index measures the amount of genetic variance that can be explained by population structures based on Wright’s F-statistics [93,94], where *F_ST_* ranges from 0 (no differentiation between subpopulations) to 1 (complete differentiation between subpopulations) [95].

### 3.6. Linkage Disequilibrium (LD)

The linkage disequilibrium (LD) values for the whole population, each subpopulation, and each chromosome were estimated by calculating the squared allele frequency correlation coefficient (*r*^2^) between each pair of SNP markers for all the distributed SNPs through the genome using the PopLDdecay software [96]. The *r*^2^ values were plotted against the corresponding genetic distances in kilobase pairs (Kbp), and a threshold value of 0.20 for *r*^2^ represents the 95th percentile of unlinked r^2^ values and was used to declare the LD decay.

## 4. Conclusions

The rapid advancements in next-generation sequencing technologies reduce the cost, time, and effort required to develop and utilize high-throughput genotyping pipelines. Using GBS technology, the current study genotyped a panel of 501 sesame accessions with 24,735 SNP markers to explore their genetic diversity and population structure. The 24K high-quality SNP markers covered 13 chromosomes, with an average 1900 SNPs/chromosome, a PIC value of 0.267, and a He value of 0.332, thus providing sufficient marker information for further studies. The population structure, PCA, and phylogenetic tree analyses identified two distinct subpopulations. The variations in polymorphism, genetic diversity indexes, and LD decay patterns indicate that directed selection and geographical adaptation may have affected the formation and differentiation within natural sesame populations at the chromosomal and, consequently, genome-wide level. This information can be used in future breeding efforts, where the genotyped panel characterized with SNP markers is a great resource for allele/gene identification using genome-wide association analysis studies (GWAS), ultimately providing a tool to enhance genetic gain in sesame breeding programs using innovative breeding methodologies such as marker-assisted selection and genomic selection.

## Figures and Tables

**Table 1:** Genomic distribution and SNP marker statistics of 24735 SNPs mapped on sesame genome using USDA sesame accessions.

Chromosomes *	Length (Mbp) *	No. of SNP	Maker Density **	
			Kbp	SNP/Mbp
LG01	23.75	2389	9.94	100.60
LG02	23.37	1240	18.85	53.05
LG03	31.44	3118	10.08	99.18
LG04	21.23	1466	14.48	69.07
LG05	20.96	1434	14.62	68.40
LG06	27.99	2467	11.35	88.13
LG07	16.12	1259	12.80	78.11
LG08	31.99	2342	13.66	73.22
LG09	26.74	2336	11.45	87.36
LG10	22.21	1705	13.03	76.76
LG11	17.33	1716	10.10	99.01
LG12	19.10	2065	9.25	108.11
LG13	19.50	1198	16.28	61.44
**Total**	301.73	24,735	-	-
**Average**	-	1903	12.76	81.73

* Chromosome names and sizes according to genome assembly; https://www.ncbi.nlm.nih.gov/datasets/genome/GCA_026168435.1/, accessed on 15 May 2024). ** Marker density as average distance between two SNPs (Kbp) and number of SNPs in 1Mbp.

**Table 2:** Percentage of transition and transversion SNPs across sesame genome using USDA sesame accessions.

SNP Type	Transitions		Transversions			
	A/G	C/T	A/T	A/C	G/T	G/C
Number of SNPs	7145	7292	2609	2555	2529	2605
Allele frequency	0.289	0.295	0.105	0.103	0.102	0.105
Total (percentage)	0.584		0.416			

**Table 3:** Analysis of molecular variance (AMOVA) among and within USDA sesame population.

Source	df	Sum of Squares	Variance Components	Variation %
Among subpopulations	1	155,958.45	308.77	29.50
Among accessions within subpopulations	499	667,180.45	599.25	57.26
Within accessions	501	69,405.00	138.53	13.24
Total			892,543.90	1046.56

**Table 4:** Linkage disequilibrium at r < 0.15 for USDA sesame accessions.

Chromosome	LD
LG01	148.77
LG02	132.90
LG03	148.88
LG04	131.75
LG05	123.26
LG06	151.14
LG07	149.29
LG08	161.50
LG09	167.65
LG10	133.18
LG11	146.04
LG12	159.01
LG13	148.82
Whole population	160.69
Subpopulation 1	166.45
Subpopulation 2	143.78

## Data Availability

Data are available upon request to the corresponding author.

## Supplementary Materials

The following supporting information can be downloaded at: https://www.mdpi.com/article/10.3390/plants13131765/s1, Figure S1: Subpopulation structure based on K = 1–10 of fastStructure software analysis; Table S1: Country and continent of origin of the 501 USDA sesame accessions used in this study, and subpopulation structure based on fastStructure software analysis.
