# Supplementary figures and images for: Genetic Diversity and Population Structure of a Large USDA Sesame Collection
Source: Plants (Basel). 2024 Jun 26;13(13):1765. doi: 10.3390/plants13131765 (PMC11243581; doi:10.3390/plants13131765)

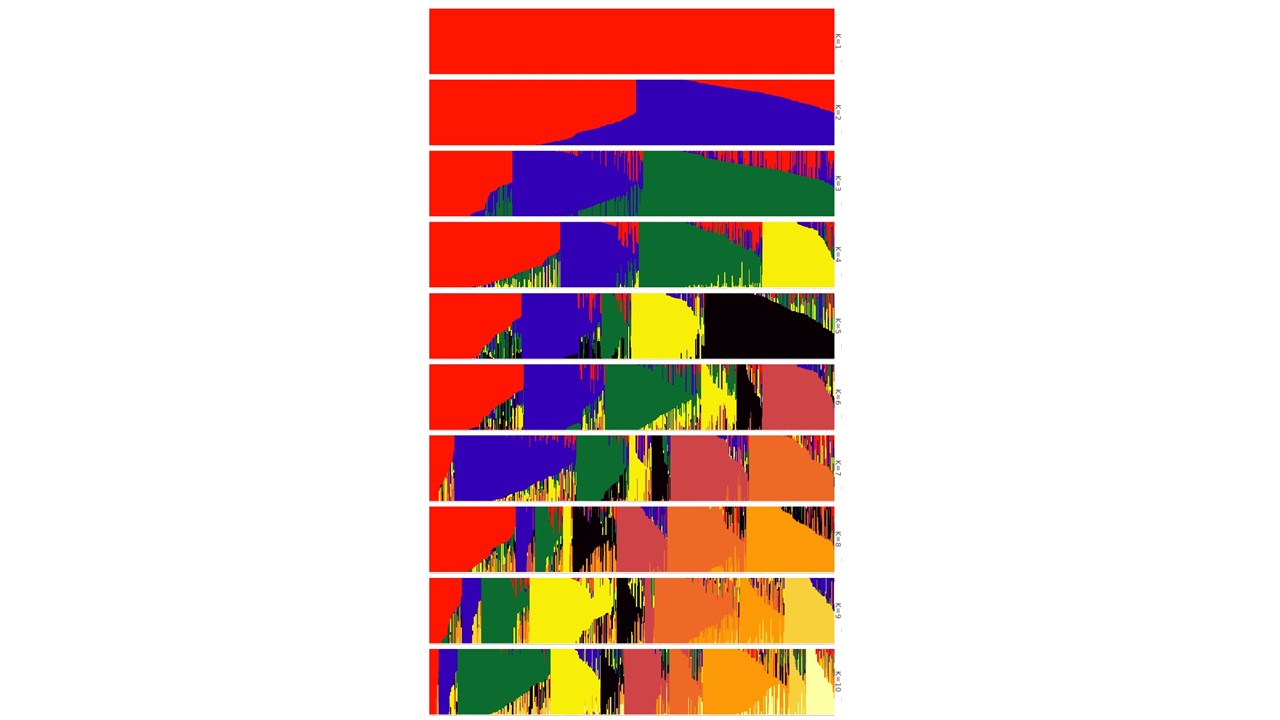

Supplement: Supplementary file 1 [file plants-13-01765-s001.zip › Figures 05042024_Supp_1.jpg]
